# Family Functioning, Identity Commitments, and School Value among Ethnic Minority and Ethnic Majority Adolescents

**DOI:** 10.1007/s10964-024-01972-1

**Published:** 2024-03-29

**Authors:** Stefanos Mastrotheodoros, Jessie Hillekens, Marta Miklikowska, Benedetta Emanuela Palladino, Francesca Lionetti

**Affiliations:** 1 Department of Youth and Family, Utrecht University, Utrecht, the Netherlands; 2 Department of Psychology, University of Crete, Crete, Greece; 3 Center for Social and Cultural Psychology, Catholic University of Leuven, Leuven, Belgium; 4 Department of Developmental Psychology, Tilburg University, Tilburg, the Netherlands; 5 Institute for Globally Distributed Open Research and Education, Gothenburg, Sweden; 6 Department of Education, Languages, Intercultures, Literatures and Psychology, University of Florence, Florence, Italy; 7 Department of Neurosciences, Imaging and Clinical Sciences, Gabriele d’Annunzio University of Chieti and Pescara, Pescara, Italy

**Keywords:** Family functioning, Identity development, School adjustment, Ethnic minority youth, Identity commitments

## Abstract

Ethnic minority youth show worse school adjustment than their ethnic majority peers. Yet, it remains unclear whether this gap can be explained by differences in family functioning and consequent identity commitments. This study examined (1) whether family functioning relates to identity commitments over time and (2) whether identity commitments impact later school value (3) among minority and majority adolescents. Minority (*N* = 205, *M*_age_ = 16.25 years, 31.1% girls) and majority adolescents (*N* = 480, *M*_age_ = 15.73 years, 47.9% girls) participated in this preregistered three-wave longitudinal study (T1: March-April 2012; T2: October 2012; T3: March-April 2013). Dynamic Panel Models revealed that most within-person cross-lagged associations were not significant in the total sample. Yet, multigroup analyses revealed differences between groups: Stronger identity commitments related to lower school value among minority adolescents, but were unrelated to school value among majority adolescents over time. Additionally, higher school value *increased* identity commitments among minority youth, yet it *decreased* identity commitments among majority youth over time. The findings highlight the differential interplay between identity commitments and school adjustment for minority and majority adolescents, with important implications for their future life chances.

## Introduction

Ethnic minority youth consistently report worse school adjustment than their ethnic majority peers, including lower school belonging and academic achievement (Heath & Brinbaum, 2014). These persistent educational inequalities have important consequences for adolescents’ future life chances and opportunities, contributing to structural inequalities later in life. Although ethnicity-based school adjustment gaps are well-documented (e.g., Heath & Brinbaum, 2014), it remains less clear which factors precede them. School adjustment refers broadly to how comfortable adolescents feel in school, how important they think school tasks are as well as their academic achievement (Demirtaş-Zorbaza & Ergeneb, 2020), and therefore comprises several subdimensions. School value refers to how valuable (vs. how boring) adolescents find attending school and doing their school tasks. Importantly, it not only precedes school belonging and academic achievement (Borgonovi et al., 2023) but also more general physical and psychological well-being (Schwartze et al., 2021). This article expands existing research by focusing on school value as a critical school adjustment outcome.

Prior research has identified several protective and risk factors in the *school* context for minority adolescents’ school adjustment, such as teacher-pupil relationships (Baysu et al., 2021) and school policies (Celeste et al., 2019). However, it remains unclear which factors in the *family* context play a role. Although adolescents spend increasingly more time with peers, they keep spending a significant amount of time in the family. Family aspects such as routines extend a long arm in adolescent and youth development (Barton et al., 2019; Brody & Flor, 1997), also influencing which peers adolescents choose to associate with (Miklikowska et al. 2019). Adolescents might discuss their school life and the importance of school with their family. Accordingly, how the family functions may impact adolescents’ commitments to choices for the future, such as further education, career, and lifestyle. Adolescents might weigh their views and possibly gain further insights during social interactions with significant others, such as family (Becht et al., 2017). Consequently, making and identifying with commitments could help adolescents cope with school-related developmental tasks, such as valuing school (Kroger & Marcia, 2011). Yet, little is known about the role of the family system functioning on minority and majority youth’s identity commitments and later school adjustment.

This preregistered, three-wave study therefore examines the longitudinal relations between family functioning, identity commitments, and school value among minority and majority adolescents. The study had a threefold aim. First, it investigates how family functioning relates to identity commitments over time. Second, it examines how identity commitments relate to adolescents’ later school value. Finally, it explores whether these aforementioned links differ between minority and majority adolescents. The term ‘minority adolescents’ will be used across different immigrant origins and migration generations to denote their minority group status in a European migration context.

### Family Functioning and Identity Commitments

Adolescents develop their identities during dynamic person-context interactions within the family. In line with a dynamic systems theory of development (Thelen & Smith, 2006), adolescent development comprises interactions between different social actors. This implies that adolescents constantly develop in relation to significant others—such as family—, but also impact them in return. The family can be seen as a dynamic system: parents impact the adolescent and their siblings, the siblings impact the adolescent and the parents, the parents impact each other, and the adolescent also impacts the other family members. These different interactions between family members lead to a constant development of all parties involved. As adolescents develop their personal identities, the family as a system must permit them to gain some personal distance from other family members to consider their options, while still maintaining connections with each other. Family members should thus be mutually differentiated during interactions to permit adolescents to develop their identities (Scabini & Manzi, 2011). Accordingly, identity development is not strictly confined within the individual, but occurs within the family as a system.

There are three aspects of the family system that the Circumplex Model of Marital and Family Systems (Olson, 2000) sees as essential for healthy family and individual member functioning: family flexibility, cohesion, and communication. Flexibility reflects leadership and organization, roles, and rules in the family. Cohesion reflects the emotional-affective pole of family relationships, such as bonding and a sense of belonging among family members (see also Scabini & Manzi, 2011). Communication describes the degree to which members openly discuss and express their views and needs (Olson, 2011). Given the importance of the family system in adolescent development, it can be expected that families that are highly flexible, cohesive, and foster open communication allow adolescents to develop a more adaptive personal identity compared to families that are rigid, enmeshed or dissociated, and have poor communication (Scabini & Manzi, 2011).

This study focuses on two specific dimensions of personal identity development, namely “commitment making” and “identification with commitments” (Luyckx et al., 2006). Commitment making reflects whether an identity choice has been made. Identification with commitments reflects how certain an adolescent feels about their identity choice (Luyckx et al., 2006). They jointly make up the ‘commitment’ aspect of identity development. Commitment is ideally reached after exploration, whereby adolescents weigh different identity options (Luyckx et al., 2006). Several studies have investigated how identity commitments develop within the context of the parent-adolescent relationship. For example, supportive parenting predicted more commitment making (Beyers & Goossens, 2008), stronger identification with commitments (Luyckx et al., 2006), and less reconsideration of commitments (Crocetti et al., 2017). Similarly, democratic and warm parenting was found to be conducive to identity development (Trost et al., 2020). Thus, empirical evidence supports the premise that parents who offer adolescents structure while being supportive create a safe environment for adolescents’ identity development. Still, parenting is only one aspect of the parent-child relationship; and the parent-child relationship is only one sub-relation of the family system. It therefore remains unclear how family functioning as a system relates to the development of adolescents’ identity commitments.

There is some cross-sectional evidence showing that adolescents with stronger identity commitments generally perceive a better family climate (Sznitman et al., 2019). More specifically, family cohesion has been positively associated with general identity commitment (Mullis et al., 2003; Rivnyák et al., 2021), as well as commitment making and identification with commitments (Prioste et al., 2020). Comparatively less is known about the associations of family flexibility with identity commitments, though some evidence suggests that youth who reported higher family flexibility tended to report lower identity commitments (Mullis et al., 2003). Regarding family communication, no study has directly assessed it along with commitment making and identification with commitments. However, tangential evidence suggests that adolescents tended to report stronger identity commitments in families with more open communication. For example, open communication was positively associated with normative identity style - an identity style that implies the presence of identity commitment processes (Bosch et al., 2012). It can therefore be argued that better family functioning could be associated with more identity commitments.

Despite this first cross-sectional evidence, research on longitudinal effects of family functioning on identity commitments is scarce. Importantly, extant cross-sectional studies cannot elucidate potential over time associations. Yet, parent-adolescent relationships (Mastrotheodoros et al., 2020a, 2020b) and family dynamics (Mastrotheodoros, 2020) change during adolescence. For example, adolescents perceive decreasing parental support (Mastrotheodoros et al., 2019) and increasing conflict intensity with their parents (Mastrotheodoros et al., 2020a, 2020b). Furthermore, at least for a notable proportion of adolescents, identity commitments increase from middle adolescence on (Becht et al., 2016). This implies that changes in parent-adolescent relationships—as one sub aspect of family dynamics—go together with changes in identity commitments. Arguably and in line with prior cross-sectional studies, changes in family functioning might also be longitudinally linked to changes in identity commitments. It is therefore important to investigate how family functioning affects identity commitments over time.

Although families comprise a core socialization context for minority and majority adolescents alike, their developmental processes may differ. In line with an integrative minority perspective in developmental science (Syed et al., 2018), minority adolescents are confronted with structural inequalities and prejudice in society that may impact their development. Although identity development is an important developmental task for all adolescents, minority adolescents face particular challenges. They navigate bicultural social worlds and learn to reconcile the culture of origin that they share with their family with the dominant culture that is shared in society at large. They must learn to embed both cultures into their sense of self as part of their identity development (Umaña-Taylor et al., 2014). At the same time, parts of their identity may be ignored or outspokenly rejected due to their minority status; and minority adolescents must juggle expectations from their family with those of peers, school, and society (Vietze et al., 2020). It is therefore important to investigate how minority adolescents’ personal identities develop and whether these processes are (dis)similar from their majority peers.

Whereas most previous research focused on majority adolescents, some studies have investigated links between family processes and identity development in minority adolescents. For example, minority parents are critical in culturally socializing their children. They discuss discrimination and bias with their children (Aral et al., 2021), as well as convey cultural values. Consequently, they are a key influence on the development of minority adolescents’ ethnic identity (Hughes et al., 2006). Interestingly, minority parents’ influence on adolescents’ ethnic identity was particularly strong in supportive parent-child relationships (Umaña-Taylor & Hill, 2020). Although barely investigated, there is evidence that siblings also influence minority adolescents’ ethnic identity development (Priest et al., 2014). These studies combined suggest that different family members discuss ethnic-racial issues with each other, shaping minority adolescents’ ethnic identity development. More closely related to the present study, minority adolescents’ family functioning was positively associated with ethnic identity belonging (Reitz et al., 2014), which reflects self-processes similar to and linked with identity commitments (Mastrotheodoros et al., 2021). Similarly, parents’ ethnic identity belonging facilitated minority adolescents’ identity development (Meca et al., 2021). Moreover, better family functioning predicted less identity confusion over time among Hispanic minority youth in the United States (and vice versa; Schwartz et al., 2009). These prior studies combined vouch for exploring the interplay of family functioning as a system and personal identity processes among minority and majority adolescents.

### Identity Commitments and School Value

Arguably, stronger identity commitments might be linked to better school adjustment outcomes. In line with ego identity development theories (Kroger & Marcia, 2011), adolescents are increasingly able to integrate different aspects of their lives into their sense of self. They learn to relate to the world around them while maintaining a stable sense of self. As they move from the exploration phase to the commitment phase, adolescents decide on their future life paths and what they want to achieve in life. Consequently, they might have a clear idea of how to reach their goal and how their education can contribute to it. This study focuses specifically on school value as one subcomponent of school adjustment (Demirtaş-Zorbaza & Ergeneb, 2020). School value can be argued to be linked to identity commitments: Adolescents might attach more value to school as a way to achieve their life goals they committed to. Still, research relating identity commitments to adolescents’ school value is completely absent. Given this scarcity, the following paragraphs refer to potential links between the broader construct school adjustment and identity commitments.

Studies taking a broader perspective suggest that stronger identity commitments are generally associated with better adjustment outcomes (see Klimstra & Denissen, 2017 for a review). For example, emerging adults with more strongly committed identities showed fewer depressive symptoms, treatment problems, emotional problems, and social problems than those with less strong identity commitments (Luyckx, Seiffge-Krenke, et al., 2008). However, prior studies have mainly focused on psychopathology symptoms and were predominantly cross-sectional (Klimstra & Denissen, 2017). One longitudinal study among Belgian university students showed that those with stronger identity commitments reported more self-esteem over time than those with weaker identity commitments (Luyckx et al., 2013). These findings point towards a positive association between identity commitments and youth’s psychosocial development. Although no prior studies examined links between identity commitments and school adjustment, psychosocial development is bidirectionally related to school adjustment (Pop et al., 2016). It could therefore be argued that stronger identity commitments could also be linked to better school adjustment over time. This study examines whether identity commitments may increase adolescents’ school value over time. Given that adolescents who show better school adjustment might also commit to an identity more easily (see Erentaitè et al., 2018 for a similar argument; Pop et al., 2016 for an empirical example), this study also examined whether school value would increase identity commitments.

Research on the interplay between identity commitments and school adjustment remains largely undocumented among both minority and majority youth. However, minority adolescents face additional challenges in their identity development and school adjustment due to their minority status (Syed et al., 2018). Interestingly, and in line with ego identity development theories (Kroger & Marcia, 2011), minority adolescents increasingly combine—or integrate—the culture of origin and the dominant culture into their sense of self with increasing age (Hillekens et al., 2019). Arguably, this development reflects increased identity commitments as minority adolescents move from an exploration phase to a commitment phase. In agreement with studies on identity commitments, minority adolescents with combined cultural identities also showed better adjustment outcomes, such as fewer internalizing problems, more life satisfaction, and better health (Spiegler et al., 2019). Additionally, minority adolescents with strongly committed ethnic identities also showed more self-esteem (Nelson et al., 2018; Sladek et al., 2020), more life satisfaction (Nelson et al., 2018), and fewer depressive symptoms (Nelson et al., 2018; Sladek et al., 2020). More closely related to the present study, a meta-analysis revealed that adolescents with more positive affect towards their ethnic origin showed better school adjustment, such as higher academic achievement and more favorable attitudes towards school (Rivas-Drake et al. 2014). Moreover, minority adolescents who combined cultural identities reported more school belonging and engagement—as related school adjustment indicators (Hillekens et al., 2023). These findings suggest that identity commitments may benefit minority adolescents’ school value as well. This study therefore explored associations between identity commitments and school value among minority and majority adolescents. Finally, there are indications that boys (Lietaert et al., 2015) and adolescents from lower socio-economic backgrounds (Sirin, 2005) show worse school adjustment compared to their peers. It is therefore important to take these factors into account to more reliably show the role of family functioning and identity commitments for adolescents’ school value as a critical school adjustment outcome.

## The Current Study

Identity commitments are expected to develop within the family context, and they are also theorized to play a role in how students experience school. Furthermore, these processes are thought to be transactional, and they can be expected to differ between adolescents with an ethnic majority and an ethnic minority status. However, the relevant literature lacks studies that test such transactional processes. The aims of this pre-registered study were threefold. First, this study examined the over-time effects of family functioning on identity commitments. It was expected that better family functioning would be associated with stronger identity commitments over time (Hypothesis 1). Specifically, it was hypothesized that links would emerge as positive within-person cross-lagged effects from family functioning to identity commitments. Second, this study investigated how identity commitments relate to adolescents’ subsequent school value. It was expected that adolescents with stronger identity commitments would show higher school value over time (Hypothesis 2). Specifically, it was hypothesized that links would emerge as positive within-person cross-lagged effects from identity commitments to higher school value (Hypothesis 2a). It was also hypothesized that positive within-person cross-lagged effects would emerge from school value to identity commitments (Hypothesis 2b). Third, this study examined whether the aforementioned processes differed between minority and majority youth; this aim was exploratory (Hypothesis 3). Finally, the current study included gender and SES as control variables.

## Method

### Sample

The sample for this study consisted of 685 high-school students, of whom 480 were majority (i.e., born in Greece and both parents born in Greece); (*M*_age_ = 15.73 years, *SD* = 0.82, 47.9% girls), and 205 were minority youth (*M*_age_ = 16.25 years, *SD* = 1.38, 31.1% girls). Of the minority adolescents, 109 (53.2%) were Albanian-origin, 47 (22.9%) were mixed-origin (one majority parent and one minority parent), and 49 (23.9%) denoted “other” as their ethnic origin. Adolescents were attending 8 high-schools in the prefecture of Athens, Attica. The schools were selected from the pool of all schools of that prefecture in order to reflect different socioeconomic strata. More information can be found in previously published studies (Mastrotheodoros et al., 2020a, 2020b).

### Procedure

Permission for the study and to access the schools was granted by the Ministry of Education of Greece. The data consisted of three waves in 12 months (two 6-months intervals), between March 2012 and March 2013. The procedures were identical in all three waves. Trained research assistants along with the first author visited the schools to inform the school principals and the teachers of the procedure and to ask permission to contact the pupils. Then, informed consent letters were sent to the pupils’ parents. Finally, adolescents were asked for their assent. Data collection took part for two 2-h teaching blocks during which adolescents were given paper-and-pencil surveys. Adolescents who did not wish to participate would stay in class for the same time.

### Variables

#### Family functioning

Family functioning was assessed using the Family Adaptability and Cohesion Evaluation Scales-IV (FACES-IV; Olson, 2011). FACES assesses several aspects of family functioning, among which flexibility (7 items), cohesion (7 items), and communication (10 items). Example items are “Our family tries new ways of dealing with problems.” for flexibility, “Family members feel very close to each other.” for cohesion, and “Family members can calmly discuss problems with each other.” for communication. Items were assessed on a 5-point Likert-scale, from 1 (*Strongly Disagree*) to 5 (*Strongly Agree*), and composite scores were constructed as means of the scale items. The FACES-IV has been adapted and tested in Greek population and has been shown to have good psychometric properties (Koutra et al., 2013; Mastrotheodoros et al., 2020a). In the current study, the scales showed acceptable internal consistencies, ranging across waves from Cronbach’s ɑ = 0.64–0.71 for flexibility, from ɑ = 0.68–0.76 for cohesion, and from ɑ = 0.88–0.90 for communication.

#### Identity commitments

The Dimensions of Identity Development Scale (DIDS) (Luyckx, Schwartz, et al., 2008) was used to assess identity commitments. The DIDS captures several aspects of identity development, among which commitment making (5 items) and identification with commitments (5 items). Example items are “I have decided on the direction I want to follow in life” for commitment making, and “Plans for the future offer me a sense of security” for identification with commitments. Items were assessed on a 5-point Likert scale, from 1 (*Strongly Disagree*) to 5 (*Strongly Agree*), and composite scores were constructed as means of the scale items. The DIDS has been translated and used in Greek language and has shown good psychometric properties (Mastrotheodoros and Motti-Stefanidi, 2017). In the current study, the scales showed good internal consistencies, ranging from Cronbach’s ɑ = 0.91 to ɑ = 0.93 for commitment making, and from ɑ = 0.83 to ɑ = 0.90 for identification with commitments.

#### School value

School value was assessed with the School Value subscale of the Educational Goals Scale (Niemivirta, 2002; Tuominen-Soini et al., 2008). It comprises three items on a 7-point Likert scale from 1 (*Not like me at all*) to 7 (*Describes me fully*), and a composite score was constructed as a mean of the scale items. Example items are “Studying is boring”, and “I feel like studying and going to school is useless”. Items were reverse-coded such that higher scores imply higher school value. The internal consistencies were acceptable in this study, ranging from Cronbach’s ɑ = 0.68 to 0.71 across the three waves.

#### Minority status

Ethnic minority background was coded as a binary variable (0—majority Greek, 1—ethnic minority).

#### Covariates

Adolescent gender and socioeconomic status (SES) were used as covariates in the main analyses. SES is a composite score consisting of adolescent-reported mother education, father education, mother employment, father employment, own house (vs. rent), and home density (ratio of people living in the same home and number of rooms in the home). The variables were measured in all three waves, resulting in three measures of SES, which were then standardized and averaged in one general SES variable across the three waves.

### Analytic Plan

The research questions, hypotheses, and analytic plan for this study have been pre-registered and can be found in the following link (https://osf.io/mpq68). First, missing data were examined using the R (R Core Team, 2020) packages *naniar* v.0.6.1 (Tierney et al., 2020) to calculate and visualize the magnitude of missing values, and *finalfit* v.1.0.4 (Harrison, 2020) to examine whether missingness depends on known variables. In this step, the skewness of the main variables was screened to decide whether a standard Maximum Likelihood or robust ML estimator would be used. Second, for family functioning and identity commitments Confirmatory Factor Analyses (CFA) were run, separately for each wave, to examine whether the structure of family functioning consisting of flexibility, cohesion, and communication, and identity commitments consisting of commitment making and identification with commitments was psychometrically sound. Third, Intraclass Correlation Coefficients were calculated for family functioning, identity commitments, and school value, to examine whether a technique that explicitly separates the between-person variance from the within-person variance was an adequate choice. Finally, to answer the research questions multiple reciprocal Dynamic Panel Models (DPM; Bollen & Brand, 2010; Dishop & DeShon, 2022) were applied: One model to address Research Questions 1 (regarding the over-time effects of family functioning on identity commitments) and 2 (regarding the over-time effects of identity commitments on school value), and two multigroup models to address Research Question 3 (regarding the differences between minority and majority youth on Research Questions 1 and 2). DPM controls for unmeasured stable covariates while taking possible bidirectional, dynamic effects into account, thereby allowing clearer conclusions regarding the links among concepts at the individual level.

### Deviations from the Pre-Registered Analytic Plan

First, it was planned to use Random-Intercept Cross-Lagged Panel Models (RI-CLPM; Hamaker et al., 2015) to analyze the longitudinal links of family functioning and identity commitments, with Grade Point Average (GPA, as a measure of school achievement) and school value, separately. However, several problems arose when modeling GPA, leading to improper solutions, including negative latent variances and out-of-bound estimates. These problems likely arose from the very high correlations (r’s > 0.85, indicating collinearity) among the repeated assessments of GPA. These problems were not overcome when fixing the error variances of these measures (e.g., see Lefcheck, 2021; also see Geiser’s suggestions in QuantFish Director, 2022a, 2022b). During the review process, reviewers asked for the modeling of a composite “school adjustment” score composed of GPA and school value, but similar improper solutions remained. Therefore, it was decided that GPA would not be modeled, and only models with school value were kept. Models including GPA are available upon request.

In addition, during the review process of this paper, several papers were published presenting the DPM (Dishop & DeShon, 2022; Murayama & Gfrörer, 2023), and comparing it to the RICLPM. These papers indicate that the DPM has some advantages over the RICLPM, such as controlling for unmeasured covariates better, allowing for accumulating effects, and a more realistic modeling of potential dynamic, causal effects (Murayama & Gfrörer, 2023). Given these advantages, the DPM was better suited to the data than RICLPM: Model fit was better and estimates were more stable. Therefore, it was decided that the reciprocal DPM as presented by Dishop and DeShon (2022) would be the model of choice.

## Results

### Preliminary Analyses

Table 1 presents the means, standard deviations, and bivariate correlations among all study variables. Table 2 presents missingness for each of the main variables. As seen there, missingness was below 30% in all cases. Examination of the missingness patterns revealed that on several of the main variables, missingness depended on socioeconomic status and/or adolescent gender. Therefore, SES and gender were controlled for in all main analyses. Regarding distributions, no main variable showed severe skewness, therefore, Maximum Likelihood estimation was applied in all main analyses.

**Table 1:** Means, standard deviations, and bivariate correlations of main variables

Variable	*M*	*SD*	1	2	3	4	5	6	7	8
1. scv1	4.96	1.39								
2. scv2	5.01	1.35	0.57**							
3. scv3	4.86	1.41	0.51**	0.61**						
4. fam1	3.51	0.59	0.19**	0.25**	0.17**					
5. fam2	3.51	0.58	0.22**	0.28**	0.23**	0.70**				
6. fam3	3.46	0.66	0.22**	0.29**	0.20**	0.66**	0.68**			
7. cmt1	3.70	0.78	0.14**	0.14**	0.16**	0.24**	0.22**	0.14**		
8. cmt2	3.86	0.74	0.15**	0.25**	0.19**	0.28**	0.34**	0.22**	0.49**	
9. cmt3	3.85	0.80	0.19**	0.26**	0.24**	0.25**	0.30**	0.26**	0.46**	0.65**

Numbers in variable names denote wave of study (Waves 1–3)

*scv* school value, *fam* family functioning, *cmt* identity commitments

**p < 0.01

**Table 2:** Frequency and relative frequency (%) of missing values for the main variables

Variable Name	N missing	% missing
scv1	129	18.8
scv2	161	23.5
scv3	153	22.3
fam1	153	22.3
fam2	173	25.3
fam3	161	23.5
cmt1	125	18.2
cmt2	88	12.8
cmt3	88	12.8

Numbers in variable names denote waves of study

*fam* family functioning, *scv* school value, *cmt* identity commitments

### Confirmatory Factor Analyses

Table 3 presents the fit indices and the standardized factor loadings of the nine CFAs to test the factor structure of family functioning, identity commitments, and school value for each of the three waves. For family functioning and school value, the models were saturated, which means that the fit indices are not informative. However, inspection of the standardized factor loadings showed that there were no loadings below the pre-registered threshold (0.30). Therefore, the factor structure of the family functioning and the school value constructs was deemed adequate to proceed with further analyses. For identity commitments, two of the three fit indices (i.e., CFI and TLI) showed very good fit. In addition, inspection of the standardized loadings showed that there is no item loading below 0.40. Therefore, both the fit indices and the standardized factor loadings indicate that the factor structure of identity commitments is good across the three waves. Finally, the Intraclass Correlation Coefficient was 0.67 for family functioning, 0.60 for identity commitments, and 0.58 for school value. Therefore, at least 10% of the variance in each measure was on the within-person level, which renders the disaggregation of the between-person from the within-person variance a viable option.

**Table 3:** Results of the Confirmatory Factor Analyses for Family Functioning and Identity Commitments

			*Model fit indices*				
	*β*	*p*	*χ*^*2*^	*df*	CFI	TLI	RMSEA
Family Functioning Wave 1			0	0	1	1	0
Flexibility 1	0.69	0.00					
Cohesion 1	0.90	0.00					
Communication 1	0.76	0.00					
Family Functioning Wave 2			0	0	1	1	0
Flexibility 2	0.69	0.00					
Cohesion 2	0.94	0.00					
Communication 2	0.77	0.00					
Family Functioning Wave 3			0	0	1	1	0
Flexibility 3	0.77	0.00					
Cohesion 3	0.89	0.00					
Communication 3	0.91	0.00					
Identity Commitments Wave 1			324	35	0.99	0.99	0.13
Item1	0.88	0.00					
Item2	0.85	0.00					
Item3	0.93	0.00					
Item4	0.82	0.00					
Item5	0.81	0.00					
Item16	0.61	0.00					
Item17	0.74	0.00					
Item18	0.74	0.00					
Item19	0.70	0.00					
Item20	0.75	0.00					
Identity Commitments Wave 2			468	35	0.99	0.99	0.16
Item1	0.88	0.00					
Item2	0.86	0.00					
Item3	0.92	0.00					
Item4	0.87	0.00					
Item5	0.89	0.00					
Item16	0.73	0.00					
Item17	0.79	0.00					
Item18	0.77	0.00					
Item19	0.73	0.00					
Item20	0.76	0.00					
Identity Commitments Wave 3			654	35	0.99	0.99	0.18
Item1	0.91	0.00					
Item2	0.89	0.00					
Item3	0.93	0.00					
Item4	0.90	0.00					
Item5	0.86	0.00					
Item16	0.81	0.00					
Item17	0.86	0.00					
Item18	0.79	0.00					
Item19	0.76	0.00					
Item20	0.81	0.00					
School Value Wave 1			0	0	1	1	0
Item 1	0.41	0.00					
Item 2	0.72	0.00					
Item 3	0.89	0.00					
School Value Wave 2			0	0	1	1	0
Item 1	0.44	0.00					
Item 2	0.78	0.00					
Item 3	0.78	0.00					
School Value Wave 3			0	0	1	1	0
Item 1	0.51	0.00					
Item 2	0.69	0.00					
Item 3	0.86	0.00					

Identity Commitments items numbering refers to the items in the Dimensions of Identity Development Scale

### Family Functioning, Identity Commitments, and School Value

**Fig. 1:**
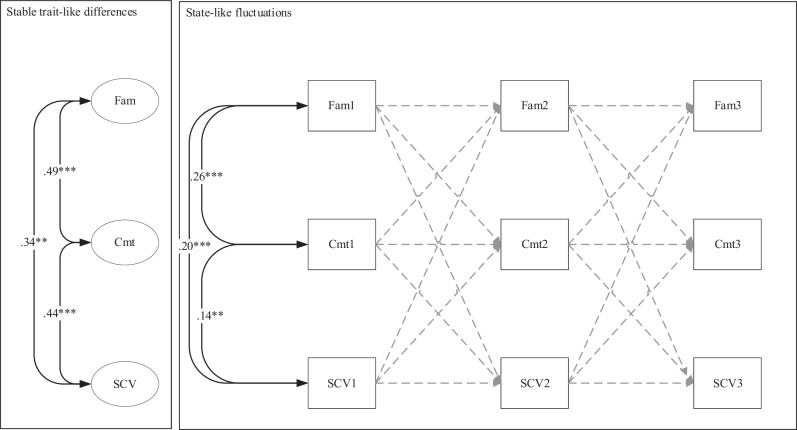
Standardized parameter estimates of the reciprocal Dynamic Panel Model, as applied by Dishop & DeShon (2022), on the dynamic associations between Family Functioning, Identity Commitments, and School Value, for the total sample. Note. Maximum Likelihood estimation was applied. Fam family functioning, SCV school value, Cmt identity commitments. *p < 0.05; **p < 0.01; ***p < 0.001

Table 4 shows the fit indices, and Table 5 all the parameter estimates of the DPM to examine the longitudinal within-person associations between family functioning, identity commitments and school value (see also Fig. 1). The fit of the overall model was good. Positive and significant between-person associations were found among the three variables. Adolescents who reported relatively higher family functioning tended to be also those who reported relatively stronger identity commitments (*r* = 0.49, *p* < 0.001), and higher school value (*r* = 0.34, *p* < 0.01), compared to other adolescents. Also, adolescents who reported stronger identity commitments tended to be those who also reported higher school value (*r* = 0.44, *p* < 0.001), compared to other adolescents. No within-person effects were found. This indicates that there are no dynamic within-person effects among family functioning, identity commitments, and school value for the total sample.

**Table 4:** Fit indices and multigroup model comparisons for all models in this study

	*χ*^*2*^ *(scaled)*	*df*	*p*	CFI	TLI	RMSEA	delta *χ*^2^
Confirmatory Factor Analysis Models							
Family_W1	0	0	0.000	1.000	1.000	0.000	
Family_W2	0	0	0.000	1.000	1.000	0.000	
Family_W3	0	0	0.000	1.000	1.000	0.000	
Commitment_W1	658	35	0.000	0.951	0.937	0.184	
Commitment_W2	754	35	0.000	0.960	0.948	0.202	
Commitment_W3	935	35	0.000	0.952	0.938	0.222	
School_Value_W1	0	0	0.000	1.000	1.000	0.000	
School_Value_W2	0	0	0.000	1.000	1.000	0.000	
School_Value_W3	0	0	0.000	1.000	1.000	0.000	
Dynamic Panel Models							
Model 1_School_Value	52.2	21	0.000	0.982	0.955	0.056	
Model 1.1_School_Value_MultiGroup_Free	73.2	42	0.002	0.985	0.961	0.052	16.91*
Model 1.2_School_Value_Multigroup_Con	90.2	51	0.001	0.978	0.953	0.056	

In the multigroup models, “Free” refers to all parameters being free to vary across majority and minority groups, whereas Con(strained) refers to all main parameters of interest (covariances, autoregressive stabilities, cross-lagged coefficients) being fixed to be equal across groups. The free model showed significantly better fit. All three Dynamic Panel Models have been controlled for gender and socioeconomic status

*p < 0.05

**Table 5:** Parameter estimates of the Dynamic Panel Model for the associations among family functioning, identity commitments, and school value for the total sample

	Unstandardized estimate	S.E.	*p*	Standardized estimate
Autoregressive Stabilities				
fam1 → fam2	−0.198	0.125	0.113	−0.203
fam2 → fam3	−0.198	0.125	0.113	−0.171
cmt1 → cmt2	0.006	0.071	0.936	0.006
cmt2 → cmt3	0.006	0.071	0.936	0.005
scv1 → scv2	−0.028	0.102	0.786	−0.029
scv2 → scv3	−0.028	0.102	0.786	−0.026
Cross-lagged Effects				
fam1 → cmt2	−0.066	0.082	0.419	−0.052
fam2 → cmt3	−0.066	0.082	0.419	−0.048
fam1 → scv2	0.134	0.153	0.379	0.060
fam2 → scv3	0.134	0.153	0.379	0.054
cmt1 → fam2	−0.008	0.034	0.815	−0.011
cmt2 → fam3	−0.008	0.034	0.815	−0.009
cmt1 → scv2	−0.168	0.096	0.080	−0.100
cmt2 → scv3	−0.168	0.096	0.080	−0.089
scv1 → cmt2	−0.011	0.027	0.686	−0.020
scv2 → cmt3	−0.011	0.027	0.686	−0.018
scv1 → fam2	0.005	0.023	0.825	0.013
scv2 → fam3	0.005	0.023	0.825	0.010
Correlations				
cmt ~~ scv	**0.304**	0.074	0.000	0.438
scv ~~ fam	**0.226**	0.075	0.003	0.335
cmt ~~ fam	**0.194**	0.046	0.000	0.486
cmt1 ~~ scv1	**0.154**	0.049	0.002	0.141
scv1 ~~ fam1	**0.166**	0.034	0.000	0.201
cmt1 ~~ fam1	**0.119**	0.024	0.000	0.258

Parameters in **bold** designate statistically significant estimates. Effects of adolescent gender and socio-economic status have been controlled for

*fam* family functioning, *cmt* identity commitments, *scv* school value. *S.E.* standard error

### Testing Differences between Minority and Majority Adolescents

Table 4 shows the fit indices and the model chi-square comparisons for the two multigroup models, one where parameters were left free to vary across groups, and another one where parameters were fixed to be equal across groups. The difference in fit was significant between the two multigroup models, indicating that the former model (free model) had better fit to the data. Table 6 presents all parameter estimates for the multigroup model with free parameters across groups.

**Table 6:** Parameter estimates of the Dynamic Panel Model for the associations among family functioning, identity commitments, and school value for the multigroup model with parameters freely estimated across groups

	Unstandardized estimate	S.E.	*p*	Standardized estimate
Minority Adolescents				
Autoregressive Stabilities				
fam1 → fam2	−0.159	0.383	0.678	−0.157
fam2 → fam3	−0.159	0.383	0.678	−0.121
cmt1 → cmt2	−0.047	0.115	0.683	−0.053
cmt2 → cmt3	−0.047	0.115	0.683	−0.040
scv1 → scv2	−0.054	0.157	0.733	−0.057
scv2 → scv3	−0.054	0.157	0.733	−0.050
Cross-lagged Effects				
fam1 → cmt2	0.185	0.157	0.238	0.144
fam2 → cmt3	0.185	0.157	0.238	0.123
fam1 → scv2	−0.014	0.316	0.964	−0.006
fam2 → scv3	−0.014	0.316	0.964	−0.006
cmt1 → fam2	−0.052	0.091	0.564	−0.075
cmt2 → fam3	−0.052	0.091	0.564	−0.050
cmt1 → scv2	−**0.426**	0.183	0.020	−0.259
cmt2 → scv3	−**0.426**	0.183	0.020	−0.213
scv1 → cmt2	**0.107**	0.050	0.033	0.212
scv2 → cmt3	**0.107**	0.050	0.033	0.168
scv1 → fam2	0.023	0.050	0.644	0.058
scv2 → fam3	0.023	0.050	0.644	0.041
Correlations				
cmt ~~ scv	0.160	0.136	0.239	0.222
scv ~~ fam	0.312	0.174	0.074	0.460
cmt ~~ fam	0.123	0.092	0.180	0.342
cmt1 ~~ scv1	**0.174**	0.085	0.040	0.155
scv1 ~~ fam1	0.092	0.057	0.106	0.120
cmt1 ~~ fam1	0.049	0.038	0.207	0.110
Majority Adolescents				
Autoregressive Stabilities				
fam1 → fam2	−0.213	0.134	0.111	−0.223
fam2 → fam3	−0.213	0.134	0.111	−0.193
cmt1 → cmt2	0.021	0.090	0.818	0.021
cmt2 → cmt3	0.021	0.090	0.818	0.020
scv1 → scv2	0.064	0.136	0.637	0.067
scv2 → scv3	0.064	0.136	0.637	0.060
Cross-lagged Effects				
fam1 → cmt2	−0.125	0.092	0.173	−0.100
fam2 → cmt3	−0.125	0.092	0.173	−0.094
fam1 → scv2	0.152	0.172	0.377	0.069
fam2 → scv3	0.152	0.172	0.377	0.062
cmt1 → fam2	0.012	0.037	0.741	0.016
cmt2 → fam3	0.012	0.037	0.741	0.014
cmt1 → scv2	−0.100	0.116	0.389	−0.058
cmt2 → scv3	−0.100	0.116	0.389	−0.054
scv1 → cmt2	−**0.065**	0.031	0.037	−0.120
scv2 → cmt3	−**0.065**	0.031	0.037	−0.113
scv1 → fam2	0.002	0.026	0.924	0.006
scv2 → fam3	0.002	0.026	0.924	0.005
Correlations				
cmt ~~ scv	**0.345**	0.088	0.000	0.546
scv ~~ fam	**0.180**	0.088	0.041	0.297
cmt ~~ fam	**0.212**	0.053	0.000	0.506
cmt1 ~~ scv1	**0.133**	0.059	0.023	0.125
scv1 ~~ fam1	**0.194**	0.043	0.000	0.231
cmt1 ~~ fam1	**0.141**	0.030	0.000	0.304

Parameters in **bold** designate statistically significant estimates. Effects of adolescent gender and socio-economic status have been controlled for

*fam* family functioning, *cmt* identity commitments, *scv* school value. *S.E.* standard error

**Fig. 2:**
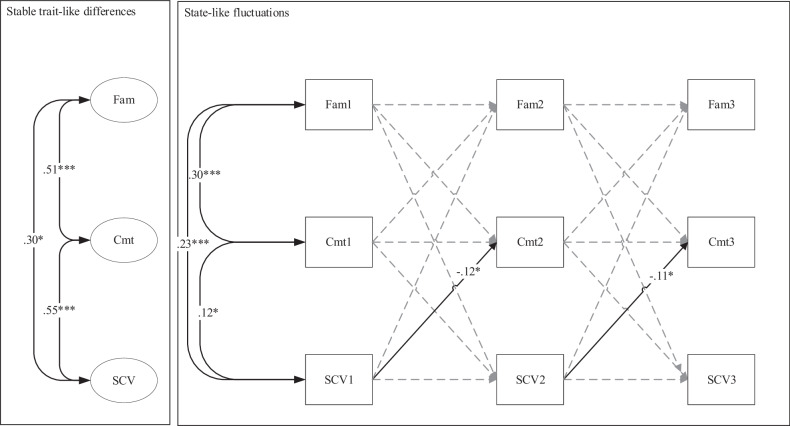
Standardized parameter estimates of the reciprocal Dynamic Panel Model, as applied by Dishop & DeShon (2022), on the dynamic associations between Family Functioning, Identity Commitments, and School Value, for majority adolescents. Note. Maximum Likelihood estimation was applied. Fam family functioning, SCV school value, Cmt identity commitments. * p < 0.05; *** *p* < 0.001

In the majority but not in the minority group, the between-person correlations between family functioning, identity commitments, and school value were positive and significant. As shown in Fig. 2, majority adolescents from families with relatively higher family functioning tended to also be those adolescents with stronger identity commitments (*r* = 0.51, *p* < 0.001), and higher school value (*r* = 0.30, *p* < 0.05), and those with stronger identity commitments tended to also be those with higher school value (*r* = 0.55, *p* < 0.001). Similar positive correlations emerged for the majority adolescents among the Wave 1 variables. For the minority adolescents, only one significant positive correlation emerged between the identity commitments and school value at Wave 1 (*r* = 0.16, *p* < 0.05).

**Fig. 3:**
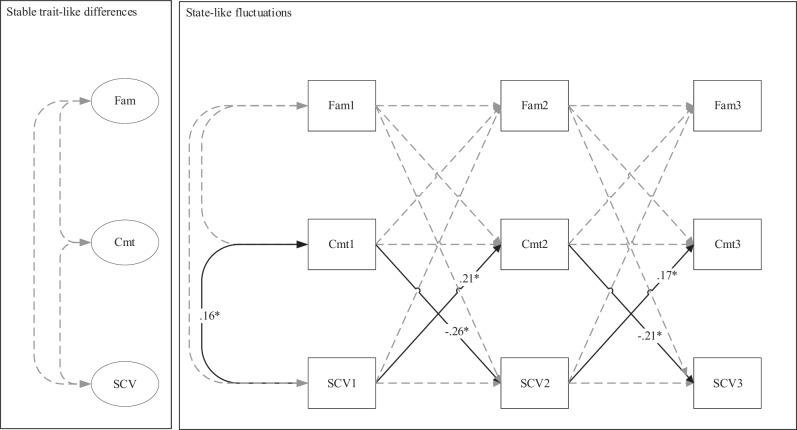
Standardized parameter estimates of the reciprocal Dynamic Panel Model, as applied by Dishop & DeShon (2022), on the dynamic associations between Family Functioning, Identity Commitments, and School Value, for minority adolescents. Note. Maximum Likelihood estimation was applied. Parameters were left free to vary across groups. Fam family functioning, SCV school value, Cmt identity commitments. * p < 0.05; *** *p* < 0.001

On the within-person level, further differences between minority and majority adolescents emerged. For majority adolescents, a negative cross-lagged effect emerged from school value to identity commitments (*β* = −0.12, *p* < 0.05 from Wave 1 to Wave 2, and *β* = −0.11, *p* < 0.05 from Wave 2 to Wave 3), indicating that increases in school value predicted *decreases* in identity commitments in majority adolescents. However, in minority adolescents, increases in school value predicted *increases* in identity commitments (*β* = 0.21, *p* < 0.05 from Wave 1 to Wave 2, and *β* = 0.17, *p* < 0.05 from Wave 2 to Wave 3). Additionally, only among minority adolescents, increases in identity commitments predicted *decreases* in school value (*β* = −0.26, *p* < 0.05 from Wave 1 to Wave 2, and *β* = −0.21, *p* < 0.05 from Wave 2 to Wave 3), indicating two opposite cross-lagged effects among identity commitments and school value only among minority youth (Fig. 3).

## Discussion

Theoretical accounts posit that family functioning, identity commitments and school adjustment can be dynamically associated with each other during adolescence, with patterns possibly differing for youth with an ethnic majority or minority background. Limited empirical evidence offers sporadic support for these relations, but more research is needed. The present study contributes to an emergent line of research examining the key role of families in youth’s identity development (Scabini & Manzi, 2011). It goes beyond previous work by examining associations between family functioning and identity development among minority and majority youth over time; and it examines critical consequences of identity development for their later school adjustment. This study drew on three-wave longitudinal data among Greek majority and minority adolescents in Greece, and applied DPM to differentiate within- from between-person processes. The findings demonstrated that within-person cross-lagged links differed between minority and majority adolescents: Whereas higher school value was associated with *stronger* identity commitments among minority youth, it was associated with *weaker* identity commitments among majority youth over time. Additionally, stronger identity commitments were associated with *lower* school value among minority adolescents over time, whereas they were unrelated to school value among majority adolescents. This study thus highlights differences in the developmental processes of minority and majority youth (Syed et al., 2018) and showcases the importance of disentangling between- from within-person developmental processes.

### Family Functioning and Identity Commitments among Minority and Majority Youth

The first aim was to examine whether better family functioning would be associated with more identity commitments over time; and to explore whether this varied among minority and majority adolescents. Across both groups, the hypothesized within-person cross-lagged (over-time) effects did not emerge (H1). Thus, experiencing higher family functioning than usual did not help adolescents to commit to their identities more strongly than usual. A possible explanation might be that the dynamic processes between family functioning and identity development might be taking place in a much shorter time scale than the one used in this study (Boele et al., 2022). Adolescent development takes place in dynamic person-context interactions. In line with a dynamic systems theory of development (Thelen & Smith, 2006), moment-to-moment interactions between different social actors lead to adolescent development. Accordingly, family dynamics might impact adolescents on a more moment-to-moment basis. For example, the interaction quality between parents and children—as one subcomponent of the family system—has been linked to moment-to-moment affect of adolescents (Bülow et al., 2022). It could be that adolescents also negotiate their identity commitments on a moment-to-moment basis. Future research could make use of more dynamic methods—such as experience sampling—to capture daily fluctuations in family dynamics in relation to identity development.

It could also be that the proposed associations would be more pronounced in late adolescence. Although identity development starts around mid-adolescence (Becht et al., 2016), it takes time for youth to commit to an identity. Developing a sense of who you are largely takes place towards late adolescence and emerging adulthood (Pasupathi & Hoyt, 2009). As adolescents grow older, they know better what is important to them and what type of person they want to be. Although youth can recognize contradicting identities around mid-adolescence, they can only resolve them around late adolescence (Pasupathi & Hoyt, 2009). Arguably, late adolescents have more opportunities to differentiate from their families while remaining in close contact, especially as they experience more autonomy than mid-adolescents. The current sample might therefore be slightly too young to detect meaningful over-time associations between family functioning and identity commitments.

Although the absence of within-person processes between family functioning and identity commitments was similar among minority and majority adolescents, some differences emerged between both groups. Specifically, there were significant positive between-person associations between family functioning and identity commitments only among majority youth: Majority adolescents from higher functioning families also reported stronger identity commitments. Better functioning families might help majority adolescents with their identity development as a key developmental task (Scabini & Manzi, 2011). They seem to provide a safe space for majority adolescents to gain personal distance while still retaining strong, positive ties among family members (Scabini & Manzi, 2011). Accordingly, majority adolescents may be better equipped to commit to an identity due to their family context. Minority youth on the contrary grow up in bicultural social worlds and learn to combine multiple cultural perspectives into their sense of self (Umaña-Taylor et al., 2014). As they negotiate their bicultural identities, they might draw from more variable sources compared to their majority peers (Hillekens et al., 2019). Whereas different socialization sources likely have similar expectations for majority youth—who grow up in monocultural worlds -, these expectations might contradict each other for minority adolescents (Vietze et al., 2020). Consequently, minority youth often have different cultural identities than their parents, which can be a source of conflict (Telzer, 2010). Another possible explanation for the absence of an association for minority youth might stem from the assimilationist context in which this study took place. In more assimilationist receiving societies (Ward & Geeraert, 2016) such as Greece, minority youth might feel that their ethnic background and their national belonging are perceived as incompatible in society (Mastrotheodoros et al., 2021). This might explain the absence of an association between family functioning (representing the ethnic background) and identity commitments (representing the future self in the national context) among minority youth specifically. It might therefore be that stronger identity commitments are associated with higher family functioning among majority youth, but unrelated with family functioning among minority youth. Future research could shed more light on the critical role of the family in identity development among minority youth compared to majority youth, by zooming in on how identity commitments might be a source of agreement or friction between family members.

### Identity Commitments and School Value among Minority and Majority Youth

The second aim of this study was to examine whether stronger identity commitments would be associated with higher school value over time; and to explore whether this varied among minority and majority adolescents. The hypothesized bidirectional within-person cross-lagged associations (H2a and H2b) between identity commitments and school value did not emerge at the total sample level, but differed between minority and majority adolescents. Specifically, this study found a *negative* within-person cross-lagged association from identity commitments to school value for minority adolescents, whereas the same association was absent among majority youth. Additionally, this study found opposite within-person cross-lagged associations from school value to identity commitments: whereas higher school value *increased* identity commitments among minority youth, it *decreased* identity commitments among majority youth.

These findings combined indicate that minority youth who value school more commit more strongly to their identity over time. Although identity commitments entail a variety of components, it is likely that especially identity components related to education and future life goals are increased (Pop et al., 2016). Among minority youth, education might be seen as a way to achieve upward mobility in order to pursue a different life path than their family. Structural educational inequalities often leave minority youth in vocational school tracks with different life prospects than their majority peers. In families who followed vocational education, school is often considered highly important as a means to further your studies to find a job and change your life prospects (Vanhalakka-Ruoho et al., 2016). In families who followed academic education, school is - on the contrary - seen as a way to pursue your dreams, rendering the opportunity to explore what one wants in life (Vanhalakka-Ruoho et al., 2016). In line with this reasoning, ethnic minority adolescents who value school more might increase their identity commitments as they perceive school as a means to an end to achieve their life goals. Ethnic majority adolescents might instead rather explore different future identities than commit to one, therefore decreasing their identity commitments over time.

Yet, European educational settings are predominantly focused on the mainstream culture, leaving little to no room for minority adolescents’ heritage cultures (Celeste et al., 2019). As such, identity components related to education (and certain lifepaths) might be rendered incompatible with minority adolescents’ ethnic identities by their majority teachers and peers. Moreover, structural barriers and prejudice in school against minority youth may pose a risk for declining school value (Baysu et al., 2018; 2021). Previous research has shown a positive link between minority youth’s national identities and school adjustment (Makarova & Birman, 2015), but inconsistent and context-dependent effects for ethnic identities (Phalet & Baysu, 2020). More specifically, in contexts where minority adolescents’ ethnic identities are not welcome or threatened, these identities have been linked to lower school adjustment. Importantly, the Greek context can be characterized as such: there is a strong pressure for minority adolescents to assimilate and relinquish the heritage culture (Motti-Stefanidi, 2023; Pavlopoulos & Motti-Stefanidi, 2017). Arguably, ethnic identities are likely also part of minority adolescents’ identity commitments, so that identity commitments might diminish school value over time only among minority youth in the current research context. Specifically, their simultaneous commitment towards educational and ethnic identities might backfire in light of structural and systemic inequalities, consequently decreasing school value over time. Future research should shed more light on the specific content of adolescents’ identity contents (e.g., educational and ethnic identities) and how they relate to subsequent school adjustment.

### Limitations and Future Directions

This study is not without limitations. First, the internal consistencies for some measures were below conventional thresholds. Since this only occurred in some waves, measures were previously validated in similar research contexts, and the confirmatory factor analyses yielded the proposed factor structure, the results were deemed robust. Second, it was not possible to differentiate minority adolescents based on their different origin groups, nor based on their generational status (1st vs 2nd + generation minority youth). Yet, experiences might vary widely. For example, some heritage cultures are more easily accepted, whereas others are perceived as more culturally distant. Adolescents from more stigmatized groups perceive more prejudice and discrimination compared to those from more accepted groups. Identity development is differentially affected due to different intergroup experiences. For example, combining ethnic and national identities is harder for adolescents from more stigmatized and racialized groups, with important implications for their school adjustment (Hillekens et al., 2023). Similarly, first generation minority youth generally attach more value to their ethnic identity compared to second and later generation peers (Birman & Trickett, 2001). Moreover, national identities might be less strong depending on the length of residence (Schwartz et al., 2015). This might make it harder to find a shared identity with peers and teachers as well. Nevertheless, minority adolescents share the experience of being a minority group member in a European migration context regardless of their origin group or migration generation. This study shows that this shared experience has implications for their developmental outcomes and takes a first step to demonstrate differential developmental processes depending on adolescents’ ethnic minority status. Still, future research should distinguish different minority groups in order to provide a more nuanced view of minority adolescents’ lived experiences.

## Conclusion

Ethnic minority youth consistently report worse school adjustment than their ethnic majority peers. Yet, little is known about which factors precede these ethnicity-based school adjustment gaps. This study used a three-wave longitudinal design and applied dynamic panel models to investigate the associations between family functioning and identity commitments as well as between identity commitments and school value among minority and majority youth. The findings showed that some within-person developmental processes differed between minority and majority adolescents. Identity processes might be more challenging for minority youth. It might be harder to juggle diverging expectations from family on the one hand, and peers and school on the other hand while developing an identity. Moreover, persistent assimilationist societal pressure makes it harder to reconcile ethnic identities with national identities such as in the Greek context that often renders both cultures to be incompatible. It is thus critical to examine which components of minority and majority adolescents’ identities contribute to their school adjustment in order to close persistent ethnicity-based school adjustment gaps. Families and schools should take the necessary steps to help minority youth to reconcile different aspects of their identities to boost the future life chances of minority youth, for example by valuing and supporting all components of their identities.
